# Overcoming the barriers to the diagnosis and management of chronic fatigue syndrome/ME in primary care: a meta synthesis of qualitative studies

**DOI:** 10.1186/1471-2296-15-44

**Published:** 2014-03-07

**Authors:** Kerin Bayliss, Mark Goodall, Anna Chisholm, Beth Fordham, Carolyn Chew-Graham, Lisa Riste, Louise Fisher, Karina Lovell, Sarah Peters, Alison Wearden

**Affiliations:** 1 Institute of Population Health, University of Manchester, Manchester, UK; 2 Institute of Psychology, Health and Society, University of Liverpool, Liverpool, UK; 3 Institute of Inflammation and Repair, University of Manchester, Manchester, UK; 4 Primary Care and Health Sciences and National School for Primary Care Research, Keele University, Keele, UK; 5 National School for Primary Care Research, University of Manchester, Manchester, UK; 6 School of Nursing, Midwifery and Social Work, University of Manchester, Manchester, UK; 7 School of Psychological Sciences, University of Manchester, Manchester, UK

**Keywords:** Chronic fatigue syndrome/ME, Barriers and facilitators, Management and diagnosis, Qualitative research, Primary health care

## Abstract

**Background:**

The NICE guideline for Chronic Fatigue Syndrome/Myalgic Encephalomyelitis (CFS/ME) emphasises the need for an early diagnosis in primary care with management tailored to patient needs. However, GPs can be reluctant to make a diagnosis and are unsure how to manage people with the condition.

**Methods:**

A meta synthesis of published qualitative studies was conducted, producing a multi-perspective description of barriers to the diagnosis and management of CFS/ME, and the ways that some health professionals have been able to overcome them. Analysis provided second-order interpretation of the original findings and developed third-order constructs to provide recommendations for the medical curriculum.

**Results:**

Twenty one qualitative studies were identified. The literature shows that for over 20 years health professionals have reported a limited understanding of CFS/ME. Working within the framework of the biomedical model has also led some GPs to be sceptical about the existence of the condition. GPs who provide a diagnosis tend to have a broader, multifactorial, model of the condition and more positive attitudes towards CFS/ME. These GPs collaborate with patients to reach agreement on symptom management, and use their therapeutic skills to promote self care.

**Conclusions:**

In order to address barriers to the diagnosis and management of CFS/ME in primary care, the limitations of the biomedical model needs to be recognised. A more flexible bio-psychosocial approach is recommended where medical school training aims to equip practitioners with the skills needed to understand, support and manage patients and provide a pathway to refer for specialist input.

## Background

Chronic Fatigue Syndrome (CFS) or Myalgic Encephalomyelitis (ME) is characterised by disabling, unexplained fatigue that is not alleviated by rest and lasts at least four months [1]. Symptoms can include headaches, unrefreshing sleep, pain, sore throat, concentration or memory problems and post exertional malaise [1]. The diagnosis is made after all relevant differential diagnoses have been excluded; the prevalence of CFS/ME among adults in both the US and the UK is estimated at around 0.2-0.4% [2]. The condition is distressing and costly in terms of both health service utilization and economic burden to patients and their families [3,4].

The UK National Institute for Health and Clinical Excellence (NICE) guideline, published in 2007 for CFS/ME, emphasises the importance of an early diagnosis [1]. However, many patients continue to experience the same numerous, complex barriers to diagnosis that were described in the 1990s [5-9]. For example, in a recent treatment trial, primary care patients reported waiting, on average, 3.7 years from onset of symptoms to diagnosis [10]. NICE guidance also recommends that patients with CFS/ME receive early treatment with the use of tailored care-packages [1]. However, 65% of members of a UK patient organisation, Action for ME, reported never receiving any treatment [11].

One possible reason for the lack of progress in the diagnosis and management of CFS/ME, is that training at medical school can engender negative attitudes and a lack of confidence in the management of CFS/ME [12]. The low status of CFS/ME is reinforced in UK practice as it is not incentivised as part of the Quality and Outcome Framework, a pay-for-performance scheme that financially rewards GP practices for achieving a number of clinical indicators [13,14].

This paper presents a meta synthesis of published qualitative studies that provide rich, bottom up data on the barriers to the diagnosis and management of CFS/ME, and the ways that some health professionals have overcome them. As the aim of the paper is to explore the experiences, values and behaviours of patients and health professionals, the authors have chosen not to include quantitative studies. This is because quantitative data does not provide sufficient insight into the reasons why some GPs are able to successfully manage CFS/ME while others are not, or the factors that help or hinder GPs within a consultation. The qualitative literature also explores issues that are external to the consultation such as policy development, taking a multifactoral approach to understanding the process of managing CFS/ME in primary care [15,16]. This paper will examine commonalities between these qualitative studies in order to make recommendations for the development of the UK medical curriculum, with the aim to improve care.

### Research question

What recommendations can be made for the development of the UK medical curriculum, the training of GPs and members of other health care professions, in order to overcome the barriers to the diagnosis and management of CFS/ME in primary care?

## Methods

Qualitative meta synthesis was used to identify interrelated themes from relevant published qualitative studies (Stages of the meta synthesis) [17,18].

Stages of the meta synthesis

1. Identifying the literature:

topic selection,

searching for studies,

appraisal of studies.

2. Data analysis and interpretation:

a. extraction of main findings from the published studies,

a. synthesis of main findings into themes to form an explanatory framework.

### Identifying the literature

**Figure 1:**
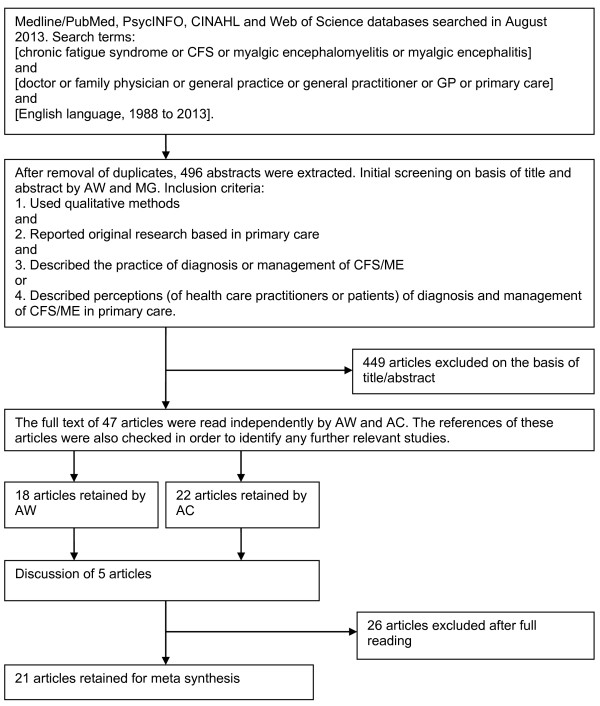
Flow diagram of search strategy and article selection.

The focus of our work was based on two substantive areas. The first was the major themes and findings related to the barriers to diagnosis and management of CFS/ME, and the second was the implications and recommendations for overcoming these barriers. A literature search was performed using Medline/PubMed, PsycINFO, CINAHL and Web of Science databases. Our search keywords were Chronic Fatigue Syndrome or CFS or Myalgic Encephalomyelitis or Myalgic Encephalitis, which were combined, using Boolean logic terms “or” and “and”, with the following list of search terms: doctor; family physician; family practice; general practice; General Practitioner; GP; and Primary Care; service. We limited our search to published English language articles and to the last 30 years of publications. The search terms, inclusion criteria and the procedure for generation of the final sample of studies are displayed in Figure 1.

After removal of duplicates, the database searches produced 496 abstracts, dated from 1988 to 2013. All abstracts were screened using the following initial questions: Is this qualitative research?; Is this research based within a primary care setting?; Does it look at patient’s beliefs and views on the diagnosis, and management of CFS/ME?; Does it examine the practitioner’s perceptions of the diagnosis, management of CFS/ME?. This produced a subset of 47 articles which were reviewed by AW and AC. Consequently, 26 articles were excluded, which although they examined the process diagnosis and management of CFS/ME within primary care, they did not report the views of either patients or practitioner to this process. In total, 21 qualitative articles were included (Figure 1). Table 1 details the characteristics of these articles.

**Table 1:** Overview of the included studies

**Study ID**	**Population (N)**	**Country setting**	**Primary objectives**	**Methods, recruitment and analysis**	**Appraisal of quality and relevance**
Asbring and Narvanen [19]	26 health professionals	Sweden	Explore physicians’ perspectives on CFS and fibromyalgia patients, specifically their thoughts about these patient groups and what strategies they use in consultations with them.	Methods: Semi-structured interviews	Relevant: 2-2-1
				Recruitment: CFS/fibromyalgia patients contacted and asked to identify physicians (patients identified through a previous study)	
				Analysis: Grounded theory principles (including constant comparison and thematic saturation but not theory development)	
Ax et al. [20]	18 (9 patients each in 2 studies)	UK (England)	Explore CFS sufferers’ accounts of patient-professional communication, patient illness beliefs and treatment expectations, and consequences of interactions regarding treatment choice.	Methods: Semi-structured interviews	Relevant: 2-1-1
				Recruitment: ME support group invitation	
				Analysis: Content analysis	
Banks and Prior [21]	114 consultations observed with patients and health professionals.	UK (Wales)	Investigate lay and professional ideas about the nature of CFS, in	Methods: Patient-professional observations in an out-patients clinic, and structured interviews with patients.	Relevant: 2-2-1
			particular, the ways in which understandings of the disorder are developed in a clinical setting.		
				Recruitment: CFS clinic (no further details)	
				Analysis: No clear description. Authors took a functional approach and analysed accounts of illness rather than beliefs about illness.	
Bayliss et al. [22]	35 (11 patients, 2 carers, 9 GPs, 5 practice nurses, 4 CFS/ME	UK (England)	Explore BME patient, health professional and community leader’s views on the barriers to the diagnosis and management of CFS/ME in the BME population.	Methods: Semi-structured interviews	Key: 2-2-2
	specialists and 5 BME community leaders)				
				Recruitment: Invited by letter/phone.	
				Analysis: Thematic analysis	
Chew-Graham et al. [7]	38 (24 patients; 14 physicians)	UK (England)	Explore how CFS patients and physicians understand the condition and how this affects the clinical consultation.	Methods: Semi-structured interviews	Key: 2-2-2
				Recruitment: Purposive sampling from participants within a previous study (physicians nominated eligible patients).	
				Analysis: Thematic analysis using constant comparison principles	
Chew-Graham et al. [23]	29 practice nurses	UK (England)	Explore practice nurses’ beliefs about CFS patients and their perceived role regarding management.	Methods: Semi-structured interviews	Key: 2-2-2
				Recruitment: Identified via involvement with a previous study and invited by letter/phone.	
				Analysis: Thematic analysis	
Chew-Graham et al. [24]	22 GPs	UK (England)	Explore GPs’ views on their role in diagnosing and managing CFS patients.	Methods: Semi-structured interviews	Relevant: 2-2-1
				Recruitment: Identified via involvement with a previous study and invited by letter/phone.	
				Analysis: Thematic analysis	
Chew-Graham et al. [25]	19 patients	UK (England)	Establish important factors for patients engaging in a CFS intervention and make recommendations for GP on referring patients to such a service.	Methods: Semi-structured interviews	Key: 2-2-2
				Recruitment: Identified GPs within a previous study and asked them to refer registered CFS patients to the study.	
				Analysis: Thematic analysis	
Clarke [26]	60 patients	Canada	Describe the way in which CFS patients seek confirmation and legitimisation of their illness.	Methods: Open-ended focused interviews	Relevant: 2-2-1
				Recruitment: Patients contacted through CFS support groups and invited to participate via letter.	
				Analysis: Cross-case analysis	
Dickson et al. [27]	14 patients	UK (Scotland)	Explore patients’ experiences of living with CFS.	Methods: Interviews	Relevant: 2-2-1
				Recruitment: Patients contacted through alternative therapy clinics (Reiki) or personal contacts.	
				Analysis: Interpretative Phenomenological Analysis (IPA)	
Edwards et al. [28]	8 patients	UK (England)	Explore the experiences of living with CFS to increase insight into the experiences of and difficulties faced by people with this condition.	Methods: Semi-structured interviews	Relevant: 1-2-2
				Recruitment: Members of an ME self-help network were recruited via posters and email.	
				Analysis: IPA	
Gilje et al. [29]	12 patients	Norway	Explore obstructions for quality care experienced by people with CFS	Methods: Group interview	Key: 2-2-2
				Recruitment: Purposive sampling from patient organisation.	
				Analysis: Systematic text condensation (Giorgi, 1985)	
Guise et al. [30]	38 patients	UK (Scotland)	Explore the interactions between health professionals and patients with CFS. Targeting sensitive issues in an online environment and exploring how the accounts were constructed.	Method: Non-directive discussion topic in an online forum.	Key: 2-2-2
				Recruitment: Patient support group invitation	
				Analysis: Discursive analysis	
Hannon et al. [5]	44 (9 GPs, 5 Practice Nurses, 4 CFS/ME specialists, 10 carers and 16 patients)	UK (England)	Explore patient, carer and health professional’s views on the development of CFS/ME training and resources for primary care.	Methods: Semi-structured interviews	Key: 2-2-2
				Recruitment: Invited by letter/phone.	
				Analysis: Thematic analysis.	
Horton et al. [31]	6 health professionals	UK (England)	Explore healthcare professionals views of best practice.	Method: Semi-structure interview with developed topic guide	Key: 2-2-2
				Recruitment: Nominated by members of England-wide study.	
				Analysis: Thematic analysis.	
Horton-Salway [32]	10 GPs	UK (England)	Explore GP’s construction of CFS/ME patient identities and the definition of their illness.	Method: Unstructured broad theme one-to-one interviews.	Relevant: 2-2-1
				Recruitment: Nominated by members of a patient support group	
				Analysis: Discourse analysis.	
McDermott et al. [33]	20 patients	UK (England)	Explore hopes and expectations of patients newly referred to CFS service (Department of Health/National Institute of Health and Clinical Excellence).	Method: Semi-structure interview with developed topic guide	Key: 2-2-2
				Recruitment: Invitation letter sent to patients newly referred to specialist CFS service by their GP.	
				Analysis: Constant comparative analysis.	
Peters et al. [34]	46 patients, 3 nurses and 2 supervisors	UK (England)	Identify potential barriers and solutions for general nurse practitioners in implementing	Method: Mixed methods nested qualitative study. Semi-structured interview with developed topic guide	Key: 2-2-2
			psychosocial interventions to people with CFS. Taken from 3 perspectives (the nurses delivering the intervention, the patients and supervisors).	Recruitment: Purposive and matched sampling (age etc)	
				Analysis: Thematic analysis.	
Raine et al. [35]	46 GPs	UK (England)	Explore GPs perspective about CFS and irritable bowel symptoms and how they should be treated.	Method: Nominal groups (clinical guideline opinion groups). Scenario evaluation.	Relevant: 2-2-1
				Recruitment: Random sample	
				Analysis: Grounded theory variant.	
Schoofs et al. [36]	16 patients	USA	Expand upon quantitative quality of life measurements to understand how healthcare (diagnosis and management) impacts upon quality of life for people with CFS and Fibromyalgia.	Method: Mixed method design. Semi-structure telephone interview.	Relevant: 2-1-1
				Recruitment: Convenience sample from 3 local support groups.	
				Analysis: Constant comparative analysis.	
Woodward et al. [37]	20 GPs and 50 patients	Australia	Compare GP and patients perspective of CFS and its management.	Method: Semi-structure interview with developed topic guide	Relevant: 2-1-1
				Recruitment: GPs recruited from Royal College. Unclear how patients were recruited.	
				Analysis: No analytic procedure defined. Mixed quantitative e.g. “50% of doctors believed…”	

### Quality appraisal

Guided by an existing quality appraisal framework that used a modified version of the CASP (Critical Appraisal Skills Programme) qualitative checklist, [38] a relevant quality appraisal tool was created by the authors and tailored to meet the aims of the meta synthesis (Appendix 1). Each paper was rated on the following criteria, using a three point scale (0 = Serious methodological issues; 1 = Minor methodological issues; 2 = Robust):

1) how relevant was the paper was to the present study’s research question?

2) to what extent did the paper add value to answering the research question?

3) and how methodologically robust was the study?

These ratings were then combined to categorise each paper as “key”, “adequate and relevant” or “flawed or not relevant” (Appendix 1). The aim was to remove any papers that were rated “flawed or not relevant” from our analysis.

Two researchers (AC and BF) carried out the quality appraisals. The researchers independently coded the remaining papers using the above criteria, thereby ensuring that all papers were double coded. Initial disagreements were successfully resolved via discussion and agreement was reached on all ratings. For example, it was highlighted that there was ambiguity around how to rate ‘relevance’ when a paper’s explicit research aim did not match the present study’s research question. Following discussion it was decided that if the findings explicitly identified ideas and concepts that answered the present study’s research question, that paper would not be penalised regarding its relevance. Furthermore it was decided that papers receiving the highest possible rating (2 for each criteria) would be categorised as key papers (Table 1). None of the 21 papers identified for analysis were classed as “not relevant”.

### Data analysis and interpretation

KB, AW and MG read and analysed each of the articles. A grid was constructed with studies along the x-axis and the content components (essential findings and interpretations) along the y-axis. Data was extracted on the original author’s analysis of the primary qualitative data. The themes derived from this primary data are called first order constructs [17,18]. A thematic approach was then taken, grouping first order constructs from each paper into core themes. The researchers recorded which papers contributed to each theme, in terms of relevant data or contradictory or contrasting results. The analysis was completed independently and then the authors met to discuss, examine and agree on emergent themes. The final core themes are termed second-order constructs as they emerge from the analysis of first order analysis of the primary data. The recommendations for practice in this meta analysis are not only consistent with the original results but also extend beyond them, considering how the second order constructs sit within the wider literature. These recommendations for the medical curriculum are termed third order constructs [17,18].

## Results

Table 1 provides an overview of the study population, the methodological strategies and the appraisal of relevance and robustness of the research. The 21 studies included in the meta-synthesis were conducted in the UK (16 studies), USA, Canada, Australia, Sweden and Norway.

The synthesis of themes is presented in two sections. The first section includes four themes which describe the barriers to the diagnosis and management of CFS/ME, and the second section outlines three themes that relate to overcoming these barriers.

Themes

1. Barriers to the diagnosis and management of CFS/ME

1.1 Illness models

1.2 The health professional-patient relationship

1.3 Knowledge and attitudes

1.4 Priorities in primary care

2. Overcoming barriers

2.1 Developing positive attitudes to CFS/ME

2.2 Developing therapeutic skills

2.3 Taking a collaborative approach

### Barriers to the diagnosis and management of CFS/ME

#### Illness models

The literature shows that both health professionals and patients tend to take a predominantly biomedical approach to understanding illness. This approach holds that symptoms are caused by underlying disease that can be measured by a definitive diagnostic test [19,22-24,26,31]. In the case of CFS/ME, the biomedical approach, which is central to the medical curriculum, leads many health professionals to conclude that there is no real illness as there is currently no identifiable pathology [7,20,23-26,31,35] while patients conclude that there must be a disease because they know there is an illness [7,21,23,31,35,38]. Despite patients experiencing real, disabling and chronic symptoms, a key paper in our analysis highlights that the scepticism among health professionals about the status of CFS/ME can lead to reluctance to make a diagnosis [7].

In primary care, it has been reported that some GPs provide a psychological label such as depression in order to avoid saying that their diagnosis is uncertain [20,22,29,31]. Other health professionals hold a “somatisation” model of illness where patients are thought to be expressing social and emotional problems in physical symptoms [19,32,37]. This approach can be experienced by patients as a blame shifting device with patients feeling held accountable for their poor health [32]. Patients may equate an interpretation of symptoms as having an origin in psychological or social problems with a belief that symptoms are imagined or fictitious and therefore reject this approach [19,20,29,31,34].

It is important for patients to feel that their symptoms are accepted and believed in order for them to engage in the management of their CFS/ME [25,27,32,34,36]. If patients are faced with a conflicting illness model, and ongoing disbelief as to the reality of their condition, they may disengage with primary care [22,29,31].

#### The health professional-patient relationship

Studies have highlighted poor communication between the patient and the health professional as a barrier to diagnosis and management of CFS/ME [7,37]. For example, GPs can fail to validate the patients’ illness experience or explain the rationale for treatment [7]. Health professionals can also be confused by the way some patients present their story [7]. Tensions are created when patients have experienced unsatisfactory consultations with previous GPs who have not provided a diagnosis. This can lead patients to be defensive or to present complicated stories with emotion or frustration [29]. Rushed consultations can also mean that patients feel unable to communicate the full extent or context of their condition [7].

Asbring and Narvanen, [19] describe how GPs can experience a lack of control in the consultation and the inability to provide a biomedical diagnosis and prescribe medication can lead to a sense of powerlessness and frustration. If a patient does not improve, practice nurses and GPs also report a lack of job satisfaction, and can distance themselves from the patient [19,23]. The breakdown of the GP-patient relationship can lead to a lack of empathetic care and patients can feel helpless and let down [7,19,28,29,35].

Bayliss et al. [22] describe how Black and Minority Ethnic (BME) patients who do not have English as their first language are not able to adequately describe their symptoms or understand the GP during a consultation. As a diagnosis of CFS/ME requires other conditions to be excluded, a number of appointments and investigations are required to reach diagnosis, which is made more difficult when communication is difficult. The term ‘Chronic Fatigue Syndrome’ can also be difficult to understand in these patient groups [22].

A poor GP-patient relationship can mean that patients turn to support groups rather than primary care for information and support. Patients with experience of CFS/ME can act as counsellors to new sufferers [20,26]. Some patients also use complementary therapy or identify types of ‘self help’ [28]. The choice of therapy was often based on recommendations from support groups [20,26].

#### Knowledge and attitudes

Raine et al. [35] report that GPs can act on their often limited understanding of CFS/ME, with little insight into what it means for the patient. Some GPs felt that their medical education had failed to equip them with the therapeutic skills necessary for diagnosing and managing patients with CFS/ME in the way recommended by current guidelines [7,19,24,27,29,33,37]. Training can also be coloured by personal opinions that senior staff hold, with patients with CFS/ME sometimes being portrayed in a derogatory manner [23,37]. A lack of knowledge about specialist services was also highlighted in the literature; with some GPs not knowing what interventions are available [25,31,38]. Patients also believed that GPs lacked the time or therapeutic expertise required to provide a sufficient level of emotional support [20,33,36].

The literature shows that some health professionals can hold inappropriately negative attitudes and stereotypical views of CFS/ME that can act as a barrier to diagnosis. For example, a number of studies have shown that some GPs believe that there are certain types of people who get CFS/ME, including those who have hypochondriasis, [36] are unmotivated, [30] pessimistic or are difficult to help [19,22,29,35]. These health professionals can believe that a diagnosis has little constructive value to patients as there are no pharmacological treatments, and that diagnosis might even become a disabling self-fulfilling prophecy [23,24,26,37]. The fact that some patients, while suffering from severe and disabling symptoms, may not look sick, can contribute to inaccurate perceptions of the condition [19].

#### Priorities in primary care

Asbring and Narvanen [19], writing in the Swedish context, suggested that CFS/ME is low ranking in the medical hierarchy as symptoms are not life threatening and it may be believed that it cannot be cured [19,29]. In the UK, health professionals also believe that there are insufficient patients with CFS/ME within their registered practice population to justify the investment of time and resources in developing and maintaining appropriate expertise [23]. Practice nurses claimed that they would be unable to develop a role in the management of patients with CFS/ME unless practices would financially benefit from this work [23]. The low status of CFS/ME in primary care is reflected in patient interviews where many describe themselves as experiencing limited medical care and attention [30].

### Overcoming the barriers

The literature shows that some health professionals have successfully diagnosed and managed CFS/ME by taking a more flexible, bio-psychosocial approach, building a positive, collaborative, therapeutic relationship with their patient. These lessons should inform the development of the medical curriculum in order to reduce inconsistencies in care.

#### Developing a more positive attitude towards CFS/ME

Health professionals have been found to change their attitude towards CFS/ME following personal experience with the condition or if they know someone with it [7,31]. Personal experience challenges stereotypical and sceptical views, and provides understanding of the impact of the condition on quality of life. GPs are then able to offer support, and may seek the knowledge required to provide a diagnosis and encourage symptom management [26,28]. This finding illustrates that attitudes can be flexible. Therefore medical school training could be more effective in engendering more positive attitudes towards the condition [5,29].

#### Developing therapeutic skills

GPs who diagnose CFS/ME placed particular importance on building a therapeutic relationship with the patient based on listening skills, respect and trust [31]. In order to provide the emotional support and information valued by patients, extended consultation times were required [5,19,37]. This allows the GP to work with the patient to prioritise the symptoms they wanted to address in order to work towards recovery [5]. Patients valued this approach, rating these GPs as particularly helpful and effective.

#### Taking a collaborative approach

GPs who diagnose CFS/ME adjust their own expectations and demands about what a physician can achieve, affirming the patients' accounts and learning from them [20,25]. Improvements may be slow and patients can experience relapse so GPs must develop resilience and understand that there is no quick fix [20]. The use of patient resources such as information sheets on how to manage the symptoms of CFS/ME can be used as a base to work with the patient to support self management [5]. Nurses also recognised the importance of investing time into explaining the rationale for the treatment and listening to and validating patients’ illness experience [34]. In doing so, they reported that they were less likely to be viewed as confronting the model of illness held by the patient, and more likely to be viewed as encouraging patients to be more active in addressing their own symptoms [31,34]. This collaborative approach is therefore beneficial in the long term management of CFS/ME as it improves communication [7]. Furthermore, successful GPs recognised that the family or significant others are instrumental in helping or hindering management [22,28]. Health professionals should therefore engage with family members where possible, as without family support, patients can feel isolated and may struggle to manage their symptoms [22,27].

## Discussion

This meta synthesis highlights a number of ways that GPs have overcome the barriers to the diagnosis and management of CFS/ME in primary care. GPs who take a therapeutic and collaborative approach are able to diagnose and manage CFS/ME in a positive way, as recommended by the UK NICE guideline [1,7,27,31]. This information is valuable in the development of the medical school curriculae and GP and health professional training to challenge the barriers that have led to the same inconsistencies in care for patients since the 1990s.

### Comparison with previous literature

In line with the findings of a review completed by Elliot [39] in the 1990s, our analysis suggests that the reluctance to diagnose and manage CFS/ME is based on scepticism and a lack of knowledge about the condition [7,23,26,31,40,41]. Edwards et al. [28] reports that a lack of training at medical school on conditions commonly called “medically unexplained” can cause frustration in the patient which leads to a breakdown of the GP-patient relationship. Larun and Malterud [15] also highlight that a diagnosis and information on the condition was necessary for recovery, as without this, the patient can feel severely ill, yet blamed and dismissed and therefore disengage. This was also a theme in the current review which found that without a functioning relationship, communication breaks down and the GP is unable to learn from the patient’s experiences or form a therapeutic relationship to manage symptoms [31]. Patients are seen as difficult and un-cooperative which compounds the negative attitudes and stereotypical beliefs held by GPs about this group. Stenhoff et al., [12] describe how such negative beliefs are then passed on to medical students, creating a new generation of doctors with the same stereotypical views about patients with CFS/ME. This maintains the barriers to the diagnosis and management of CFS/ME that have existed for the past 20 years [12].

### Implications for practice

The barriers to the diagnosis and management of CFS/ME highlighted in this study result in a significant burden of dissatisfaction for both patients and health professionals [5,24,42,43]. However, at least in the UK, the expectation that CFS/ME will normally be diagnosed and managed within primary care means that GPs need to find a way to engage with these patients [5].

This meta synthesis highlights that only a minority of health professionals report that they are able to successfully diagnose and manage patients with CFS/ME in primary care. This finding suggests that there is a need to address the inadequacy of medical training about this condition [5,10,15,19,23,24,31,35]. For example, the somatisation model, used by many GPs to explain the symptoms of CFS/ME, arises out of the failure of the biomedical model [19,32,37]. It comes from reasoning that if there is no disease underlying the illness, the illness must be the manifestation of emotional distress. The bio-psychosocial model may be a better starting point for explaining and teaching medical students about conditions such as CFS/ME. This approach suggests a multi-factorial explanation for the condition, with interactions between biological, psychological and social factors maintaining symptoms. It focuses on a patient-centred consultation, using the collaborative and therapeutic skills shown to be valuable in this meta synthesis, to develop rapport and trust with a patient, listening and validating their experiences. Once the patient feels understood the GP can then go on to discuss the management of the condition [44]. CFS/ME therefore needs to be presented to students in a positive way, with clear and simple messages [45,46]. This could mean teaching students about these conditions first before they start to habitually use the biomedical model as a prototype for all conditions. Stenhoff et al. [12] state that the first step in achieving this change is to address potential negative attitudes among trainers as sceptical attitudes towards CFS/ME can be learned by students.

An additional problem in the diagnosis and management of CFS/ME is the persistence of the term “medically unexplained illness”, which indicates diagnoses can only be by exclusion and does not allow for a positive diagnosis. This can provide a reason for medical professionals to delay diagnosis and even opt out of treatment as they feel that they have nothing to offer [47]. A fundamental shift in the perceived role of the GP is required to enable the holistic and therapeutic approach necessary to diagnose and manage CFS/ME in a positive way, as recommended by the NICE guideline [1].

### Limitations

The aim of this study was to systematically describe other interpretative studies. However, the varying research designs and level of detail provided in the qualitative studies (Table 1) make it difficult to objectively synthesise results across every study. The synthesis also only found studies from developed countries (UK, USA, Canada, Australia, Sweden and Norway), which used convenience sampling in primary care settings, CFS/ME support groups, and patient organizations. It is possible that there are other relevant studies that were not identified by our search terms. In order to address issues of consistency of reporting for this metasynthesis, the authors focused on peer reviewed, published papers only. The grey literature was therefore not included.

Formal validation of the novel quality appraisal tool used was not conducted which may have led to bias within quality coding judgements, However, double coding procedures were used to enhance the reliability of coder judgements thereby reducing any subjectivity which had potential to influence the results.

A number of the authors of this paper were involved in some of the original studies included in the synthesis. This is an inevitable finding from the limited number of researchers working in the field. In order to reduce bias, four researchers who were not authors of any of the original studies were involved in the reviewing, data extraction and analysis stages.

## Conclusions

This meta synthesis shows that some GPs have overcome the multiple barriers to the diagnosis and management of CFS/ME by building a positive, collaborative relationship with the patient, taking time to explain the rationale for treatment and validating the patients’ illness experience. However, our analysis highlights that this good practice has not been adopted by all health professionals, and the same barriers to diagnosis and management reported in the 1990s continue to be a problem today.

In order to learn from the success stories, the literature suggests the need to broaden the understanding of what the practice of medicine entails. This includes developing the curriculum to help health professionals to consider that the biomedical model, in which illness is understood in terms of underlying disease, is not appropriate for all conditions, and to understand that patients may experience real, disabling and chronic symptoms without an identifiable underlying pathology. We suggest that by teaching a more flexible, biopsychosocial approach to understanding illness, coupled with a focus on therapeutic skills to support symptom management, medical trainers may ultimately produce practitioners who are better able to diagnose, engage with and manage patients with CFS/ME, and also understand when to refer for specialist management.

## Appendix 1

CFS Meta-synthesis Quality Appraisal Form

***Notes for researchers***. For each article answer the questions below and make notes illustrating evidence from articles to support your judgements. Following this, assign each article a global category based on these responses. Categories are hierarchical and include the following:

**Key papers**: receive a score of 2 for all questions (i.e. 2-2-2).

**Adequate and relevant papers**: receive a score of 2 or 1 for any question (i.e. 2-2-1, 1-2-1 etc.).

**Flawed or not relevant papers**: receive a score of 0 for any question (i.e. 0-1-1).

Question 1: Relevance

How relevant is the article in relation to the review’s research question? (Consider to what extent the article is able to address the research question and how much of the article is focused upon this)

0 = Not at all relevant

1 = Somewhat relevant

2 = Very relevant

Evidence from article:

Question 2: Value

What is the value of the article in terms of contributing to addressing the research question? (Consider how insightful the findings are to this field of research; and how novel/original/detailed the findings are)

0 = Contributes no/very little value

1 = Contributes some value

2 = Contributes a lot of value

Evidence from article:

Question 3: Methodological robustness

How methodologically robust is the study? (Consider suitability of research design/analysis; depth of analysis; potential for bias)

0 = Serious methodological issues

1 = Some methodological issues

2 = Methodologically robust

Evidence from article:

## Competing interests

The authors declare that they have no competing interests.

## Authors’ contributions

KB contributed to the literature search, read and analysed each of the articles and drafted the paper. MG contributed to the literature search and read and analysed each of the articles. AC and BF completed the quality appraisal and contributed to the writing of the paper. CCG, LR, LF, KL and SP contributed to the research design and the writing the paper. AW designed and managed this meta synthesis. She contributed to the literature search, quality appraisal, analysis and writing of the paper. All authors read and approved the final manuscript.

## Pre-publication history

The pre-publication history for this paper can be accessed here:

http://www.biomedcentral.com/1471-2296/15/44/prepub
